# Rapid atrial pacing induces myocardial fibrosis by down-regulating Smad7 via microRNA-21 in rabbit

**DOI:** 10.1007/s00380-016-0808-z

**Published:** 2016-03-11

**Authors:** Xuyu He, Kunyi Zhang, Xiuren Gao, Liwen Li, Hong Tan, Jiyan Chen, Yingling Zhou

**Affiliations:** 1 Department of Cardiology, Guangdong Cardiovascular Institute, Guangdong Provincial Key Laboratory of Coronary Disease, Guangdong General Hospital, Guangdong Academy of Medical Sciences, 106 Zhongshan Road 2, Guangzhou, 510080 China; 2 Sun Yat-sen University Cancer Center, Sun Yat-sen University, Guangzhou, 510060 China; 3 State Key Laboratory of Oncology in South China, Collaborative Innovation Center for Cancer Medicine, Sun Yat-sen University, Guangzhou, 510060 China; 4 Department of Radiation Oncology, Cancer Center, Sun Yat-sen University, Guangzhou, 510060 China; 5 Department of Cardiology, First Affiliated Hospital, Sun Yat-sen University, Guangzhou, 510080 China

**Keywords:** Atrial fibrosis, Atrial fibrillation, Smad7, MicroRNA-21, Transforming growth factor-β_1_

## Abstract

Tachycardia-induced atrial fibrosis is a hallmark of the structural remodeling of atrial fibrillation (AF). The mechanisms underlying tachycardia-induced atrial fibrosis remain unclear. In our previous study, we found that Smad7-downregulation promoted the development of atrial fibrosis in AF. Fibroblasts are enriched in microRNA-21 (miR-21), which contributes to the development of fibrosis and heart failure in the cardiovascular system. Our study was designed to test the hypothesis that miR-21 reinforces the TGF-β_1_/Smad signaling pathway in AF-induced atrial fibrosis by down-regulating Smad7. Rapid atrial pacing (RAP, 1000 ppm) was applied to the left atrium of the rabbit heart to induce atrial fibrillation and fibrosis. qRT-PCR and northern blot analysis revealed that RAP caused a marked increase in the expression of miR-21. Transfection with a miR-21 inhibitor significantly increased the expression of Smad7, while the expression of collagen I/III significantly decreased. These changes were implicated in the AF-induced release of miR-21 and down-regulation of Smad7. Adult rat cardiac fibroblasts treated with TGF-β_1_ showed increased miR-21 expression and decreased Smad7 expression. Pretreatment with a TGF-β_1_ inhibitor reduced the TGF-β_1_-induced up-regulation of miR-21. Pretreatment with pre-miR-21 and a miR-21 inhibitor significantly decreased and increased Smad7 expression, respectively. This result was negatively correlated with the expression of collagen I/III in fibroblasts. Moreover, the results of a luciferase activity assay suggest that Smad7 is a validated miR-21 target in CFs. Our results provide compelling evidence that the miR-21 specific degradation of Smad7 may decrease the inhibitory feedback regulation of TGF-β1/Smad signaling and serves as a new insight of the mechanism of atrial fibrosis in atrial fibrillation.

**Electronic supplementary material:**

The online version of this article (doi:10.1007/s00380-016-0808-z) contains supplementary material, which is available to authorized users.

## Introduction

Atrial fibrillation (AF) is one of the most common cardiac arrhythmias and remains a major contributor to morbidity and mortality in clinical practice [1, 2]. Myocardial fibrosis plays a key role in the development and maintenance of AF [3, 4]. A better understanding of the mechanisms underlying myocardial fibrosis in AF may contribute to the more effective prevention and treatment of AF in the future.

Myocardial fibrosis has been proved to be the main cause of atrial fibrillation [3, 5]. Atrial fibrosis causes intra- and inter-atrial inhomogeneity in conduction, thus creating a substrate for local re-entry and contributing to the maintenance and progression of AF [6–8]. Our previous study clearly indicated that atrial fibrosis plays an important role in the induction and perpetuation of AF [9, 10]. The transforming growth factor-β_1_ (TGF-β_1_)/Smad signaling pathway is one of the most classic signaling pathways involved in the regulation of fibrosis [9, 10]. In the pathological conditions of AF, TGF-β_1_ synthesis and secretion is increased [3, 10, 11]. Activated TGF-β_1_ binds to membrane-bound heteromeric receptor kinases (TβRI and TβRII) that transduce intracellular signals via both Smad and non-Smad pathways. Activated TβRI and TβRII receptors phosphorylate receptor regulated Smads (R-Smads, such as Smad2/3), which form homomeric complexes and heteromeric complexes with a co-Smad (Smad4) [8, 9, 12]. These activated Smad complexes translocate into the nucleus, where they accumulate and bind to target genes to directly regulate their transcription and the production of related proteins such as fibrin [5, 8]. The inhibitory Smad proteins (I-Smads, such as Smad6/7) are capable of inhibiting TGF-β_1_ signaling. Smad7 binds to activated TβRI and prevents the phosphorylation of Smad2/3 or recruits the ubiquitin ligases Smurf1 and Smurf2 to induce proteasomal degradation of the receptor complexes. Therefore, Smad7 may act as a major negative regulator, forming autoinhibitory feedback loops in the TGF-β_1_/Smad2/3 signaling pathway [11, 12]. We previously reported that the high expression of TGF-β_1_ in pathological conditions could cause the down-regulation of Smad7, which contributes to the development of myocardial fibrosis in AF [10]. However, the molecular mechanism of the regulation of Smad7 expression through TGF-β_1_ remains unclear in AF.

MicroRNAs (miRNAs) are a broad class of small non-coding RNAs that control the expression of complementary target messenger RNAs. MicroRNA-21 (miR-21) is consistently reported to be upregulated in both cancer and various forms of cardiovascular disease [13, 14]. Fibroblasts in the cardiovascular system are enriched in miR-21, which contributes to the development of fibrosis and heart failure [15]. miR-21 has therefore emerged as an interesting candidate for the development of therapeutic strategies against many forms of heart disease. The inhibition of miR-21 by synthetic miRNA antagonists improved heart function in a cardiac disease model [16–19]. The same beneficial effects were observed in miR-21 knockout mice subjected to pressure-overload of the left ventricle, underlining the potential of miR-21 as a therapeutic target [20]. In recent years, additional roles of miR-21 in cardiovascular and pulmonary diseases have been described, including cardiac and pulmonary fibrosis as well as myocardial infarction [21–23]. It has been reported that TGF-β_1_ can increase miR-21 expression in cardiac fibroblasts and may be involved in the regulation of myocardial fibrosis [22, 24]. Animal experiments found that miR-21 expression affected the TGF-β_1_-induced renal tubular epithelial-to-mesenchymal transition in diabetic nephropathy by regulating Smad7 [25]. However, mechanistic data on the relationship between miR-21 and the TGF-β_1_/Smad7 signaling pathway in AF are still missing. In the present study, our aim was to determine if miR-21 up-regulation could enhance TGF-β_1_-induced myocardial fibrosis by inhibiting Smad7.

## Materials and methods

### Ethical standards

This study complied with the Declaration of Helsinki and the ethics committee of Guangdong Cardiovascular Institute, Guangdong General Hospital approved the study protocol. Informed consent from all participants was obtained before the initial coronary angiography. All of the experimental procedures were performed according to the Guide for the Care and Use of Laboratory Animals (NIH Publication No. 85-23, revised 1996) and were in compliance with the guidelines specified by the Chinese Heart Association policy on research animal use and the Public Health Service policy on the use of laboratory animals.

### Animals

Male New Zealand rabbits (2.0–2.5 kg) were randomly divided into 4 groups (*n* = 10 for each group): normal control (CR), sham control (SH), rapid atrial pacing (RAP) and RAP + miR-21 inhibitor(the miR-21 inhibitor was administered to this group through a lentiviral vector, Genechem, Shanghai, China). All of the rabbits except for one in the RAP group survived to the end of the study. The data generated from the dead rabbit was not included in the results presented in this paper.

### Animal model and experimental design

All the rabbits were intravenously anesthetized with 30 mg kg^−1^ pentobarbital sodium before being intubated and placed on mechanical ventilation with a volume-cycled ventilator (Model HX-200, TAIMENG, Chengdu, China). The heart was exposed at the center of the breastbone and a custom-designed set of electrodes, comprising a pair of electrodes with a distal hook for pacing and a pair of electrodes was sutured to the epicardial surface of the left atrium. The electrodes contain an interelectrode distance of 15 mm aligned proximally that serves for recording. The distal ends of these electrodes leads were tunneled subcutaneously and exposed at the animal’s back, where the electrodes were then connected to a pacemaker (output of 6 V with 1.0 ms pulse duration, Guangzhou Academy of Sciences, China) in the jacket. The pacemaker was programmed to provide RAP at 1000 ppm, and this pacing rate was maintained continuously for 4 weeks with a brief breaking period for the measurement of electrophysiological and mechanical parameters (Supplementary Table 1) [10, 26]. The rabbits in Group CR were not subjected to surgery while those in Group SH (sham control) experienced identical surgical procedure, but no RAP. When surgery was completed, the rabbits were given antibiotics and allowed to recover for 5 days. Postoperative care included the administration of antibiotics and analgesics.

MiR-control lentivirus vectors (109 TU/ml, 4 μl/day) and miR-21 inhibitor lentivirus vectors (109 TU/ml, 4 μl/day) were injected 2 times/per week for 4 weeks. The transcript for the miR-21 inhibitor was PCR purified and inserted into the lentiviral vector under the control of the CMV promoter. The GFP reporter gene was inserted at the 3′ end of the miR-21 inhibitor gene. Lentiviral vectors pseudotyped with the VSVg coat were produced. One day prior to pacing, the miRNA lentiviral vectors were injected into the jugular vein. At the end of the experiment, all of the rabbits were euthanized, and the left atria were examined histopathologically and collected for mRNA and protein analysis.

### Masson trichrome staining for collagen

Masson trichrome staining of the paraffin section prepared from the Bouin-fixed samples was performed as previously described [27]. Adobe Photoshop 5.5 and Scion Images for Windows Beta 4.0.2 software were used for quantitate atrial collagen content [25].

### Cell culture and transfection

The experimental procedure to isolate cardiac fibroblasts was approved by the Animal Care Committee of Guangzhou, China and was performed according to the method of Meszaros et al. Briefly, the ventricles of 2–3 hearts from adult male Sprague–Dawley rats (250–300 g) were minced, pooled, and placed in a collagenase/protease (Sigma, St Louis, MO, USA) digestion solution. The cells dissociated in the first treatment were discarded. After three digestions lasting 6 min each, the cells were pooled. The debris and myocytes were removed by unit gravity sedimentation. The fibroblasts were suspended in Dulbecco’s Modified Eagle Medium (DMEM) containing 1 % penicillin/streptomycin and 10 % fetal bovine serum. After a 60-min period of attachment to uncoated culture plates, those cells that were weakly attached or unattached were rinsed free and discarded. The attached cells were cultured in an incubator with 5 % CO_2_ at 37 °C. The culture medium was changed every other day. These cultures contained N95 % cardiac fibroblasts (CFs) as indicated by positive vimentin expression and negative myoactin expression. Those cells in passages 2 and 3 were used in the following studies.

Cells were plated on 60 mm^2^ plates at a density of 1 × 10^6^ cells per plate. When the cells reached 70–80 % confluency, they were transfected with pre-miR-21 or control pre-miR (Ambion, Austin, TX, USA) using the TransIT TKO transfection reagent (Mirus Bio, Madison, WI, USA). To investigate the role of miR-21 in TGF-β_1_-induced collagen expression, we performed miR-21 transfection experiments in CFs. For these experiments, the cells were seeded at a density of 2 × 10^4^ cells/cm^2^ in serum-free DMEM/F12. In this study, the cells were divided into the following groups: cells without transfection (blank control group, Group CR), cells transfected with the miR-control lentiviral vector (30 μM) (miR-control group, Group M), cells transfected with the pre-miR-21 (miR-21 over-expression) (30 μM) lentiviral vector (pre-miR-21 group, Group PM), cells treated with 10 ng/ml TGF-β_1_ (TGF-β_1_ group, Group T), cells treated with 10 ng/ml TGF-β_1_ plus the pre-miR-21 vector (30 μM) (TGF-β_1_ + pre-miR-21 group, Group TP), and cells treated with 10 ng/ml TGF-β_1_ plus the miR-21 inhibitor (30 μM) (TGF-β_1_ + miR-21 inhibitor group, Group TI).

### Real-time PCR

Total RNA was extracted with TRIzol reagent (Gibco-BRL Life Technologies, New York, USA). cDNA was synthesized with the SYBR ExScript RT-PCR kit (TOYOBO, Japan) according to the protocol provided by the manufacturer. PCR primers for TGF-β_1_ (forward: 5′-ACA TTG ACTTCC GCA AGG AC-3′; reverse: 5′-TAG TAC ACG ATG GGC AGTGG-3′), Smad7 (forward: 5′-GTG GCA TAC TGG GAG GAGAA-3′; reverse: 5′-GAT GGA GAA ACC AGG GAA CA-3′), collagen I (forward: 5′-TGG CAA GAA CGG AGA TGAC-3′; reverse: 5′-TCC AAA CCA CTG AAA CCT CTG-3′), and collagen III (forward: 5′-TTC CTT TGT GGG CTG TGT CT-3′; reverse: 5′-TTG GCT TCT CTC ACT TTC CAG-3′) were designed with Primer Express software (Applied Biosystems). GAPDH (forward: 5′-GCA CCG TCA AGG CTG AGA AC-3; reverse: 5′-ATG GTG GTG AAG ACG CCA GT-3′) was used as a reference to normalize the input amount of RNA for all samples. Real-time PCR was performed using an ABI7300 Real-Time PCR system (Applied Biosystems) with the SYBR green fluorophore. All of the reactions were performed in at least duplicate for every sample. Threshold cycle (Ct) data were collected using the Sequence Detection Software version 1.2.3 (Applied Biosystems). The fold change in mRNA level relative to GAPDH was calculated using the 2^∆∆Ct^ method [28].

TaqMan MicroRNA Reverse transcription reactions and TaqMan MicroRNA quantitative polymerase chain reactions (qPCR) were performed to detect miR-21 and an endogenous control, RNA U6 small nuclear (RNU6B) expression using the MicroRNA TaqMan Reverse Transcription Kit and the TaqMan MicroRNA Assays (Applied BioSystems, Carlsbad, CA, USA) according to manufacturer’s instructions. The miRNA detection conditions were: 95 °C for 10 min and 40 cycles of 95 °C for 15 s, 60 °C for 1 min. The miRNA expression levels were calculated as the cycle threshold (-delta CT) of miR-21 and normalized with an endogenous control. MiR-21 (forward: 5′-GCA CCG TCA AGG CTG AGA AC-3; reverse: 5′- CAG CCC ATC GAC TGG TG-3′) and RUN6 (forward: 5′-CTC GCT TCG GCA GCA CA-3; reverse: 5′- AAC GCT TCA CGA ATT TGC GT-3′) were used as a reference to normalize the input amount of RNA for all samples. Real-time PCR was performed using an ABI7300 Real-Time PCR system (Applied Biosystems) with the SYBR green fluorophore. All of the reactions were performed in at least duplicate for every sample. Threshold cycle (Ct) data were collected using the Sequence Detection Software version 1.2.3 (Applied Biosystems). The fold change in mRNA level relative to GAPDH was calculated using the 2^∆∆Ct^ method [29].

### Northern blotting

RNA was extracted by standard trizol method and suspended in DNAse- and RNAse-free water. Samples were run on 15 % 7 M urea-acrylamide gels. Electrophoresis was performed at 20 mA for about 3 h at room temperature. The oligonucleotides to be used as probes were purchased from Sigma (Milan, Italy). After electrophoresis on urea-acrylamide gels, the RNA was transferred to hybond nylon filters at 10 V overnight at 4 °C. After the ultraviolet cross-linking, the filters were prehybridized in 6 X SSPE, 5 X DENHARDT and 0.5 % SDS solution. Ten picomoles probes were radiolabelled using P^32^ γATP by standard reaction at 37 °C. Microspin G25 columns (Amersham) were used to separate radiolabelled probe from unincorporated γATP. The prehybridization and hybridization were performed in the same buffer, at 37 °C overnight. The filters were washed once in prewarmed washing solution containing 6 X SSPE and exposed in cassettes containing phosphor screen overnight. The signals were read by Amersham Typhoon 9200 phosphoimager and densitometry was performed with IMAGEQUANT software (Amersham). The filters were stripped in a solution containing 0.2 × SSPE and 0.2 % SDS for 10 min at 95 °C and subsequently rehybridized with another probe [30].

### Luciferase reporter gene assays

To examine whether Smad7 is a valid target of miR-21, a single copy of the putative miR-21-recognition element from the 3′-UTR of the Smad7 gene was cloned into the GV306 plasmid vector downstream of the dual luciferase reporter gene (Genechem, Shanghai, China). CFs were co-transfected with the GV306 vector containing the Smad7 3′-UTR and the miR-21 overexpression plasmid using Lipofectamine 2000 (Invitrogen, Carlsbad, CA, USA). Co-transfection with a non-targeting negative control RNA was performed as a control. At 48 h post transfection, the cells were lysed and assayed for luciferase activity with a dual-luciferase reporter assay kit (Promega, Madison, WI, USA) on a luminometer (Lumat LB9507).

### Immunohistochemistry

After fixed in 10 % neutral formalin for 24 h, the middle ring of left atrium was embedded in paraffin and cut into 4 μm thick sections. The section was incubated with primary antibodies (goat polyclonal anti-Smad7 antibody from Santa Cruz Biotechnology at a 1:150 dilution) at 4 °C overnight. Incubation with the secondary antibody (biotinylated rabbit anti-goat antibody also from Boster Biotect CO). was performed at 37 °C for 30 min. Staining was generated with the streptavidin biotin-peroxidase complex immunohistochemical staining kit from Boster Biotect CO. Negative controls were performed by leaving out the primary antibody. Sections were viewed with 400× magnification and the intensity of the collagen staining was analyzed using the IBAS2.5 Image Analytical System (Institute of Biomedical Engineering, Beijing, China) as described before (Sheng, 1995). Five random fields per section were analyzed to produce a single number for the section [31].

### Immunocytochemistry

The experimental procedure was performed as described previously [9], the cells were incubated with a primary antibody (anti-vimentin, myoactin, or Smad7) (1:100) in 5 % BSA overnight at 4 °C. The cells were then washed and incubated with a rhodamine-conjugated secondary antibody (1:500). The intensity of the Smad7 staining was analyzed using the IBAS2.5 Image Analytical System (Institute of Biomedical Engineering, Beijing, China) as previously described [10].

### Western blot

Western blot was performed as described previously [12]. The primary antibodies (Santa Cruz Biotechnology) were diluted as follows: TGF-β_1_, 1:200; Smad7, 1:250; collagen I, 1:200; collagen III, 1:200; and β-actin, 1:500. Horseradish peroxidase–labeled secondary antibodies (Cell Signaling Technology) were diluted 1:1000 with 0.2 % TBS-T and 1 % skim milk. The protein bands on the Western blots were visualized using ECL Plus (Amersham, Arlington Heights, IL, USA). The relative band densities were normalized against β-actin.

### Statistical analysis

The data are expressed as mean ± SE. ANOVA and the Student’s *t* test were used to determine statistical significance. A two-tailed probability value of 0.05 was considered statistically significant.

## Results

### MiRNA-21 expression profile in atrial tachypacing-induced rabbit model of AF

**Fig. 1:**
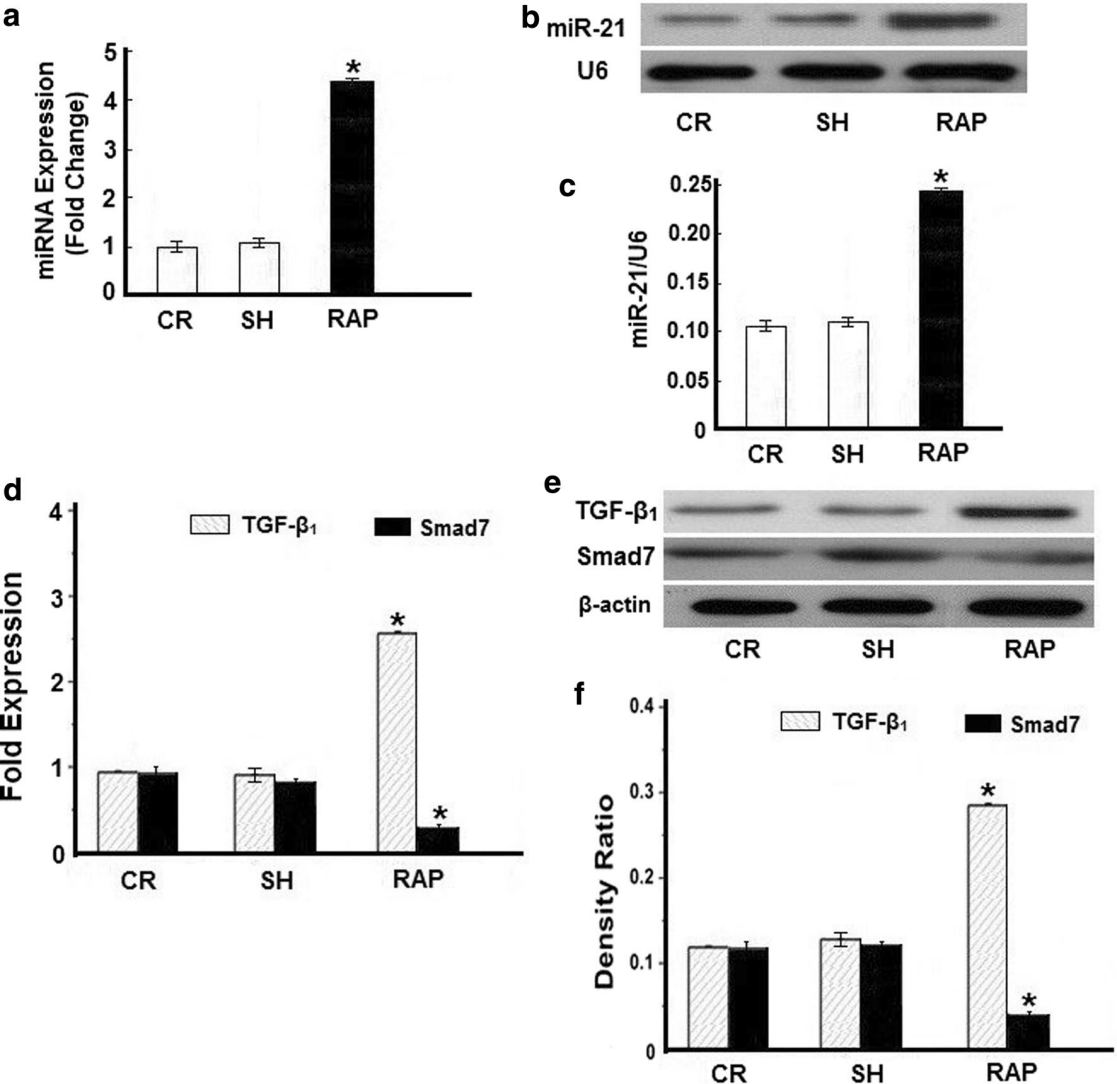
MiRNA expression profiling in atrial samples from a rabbit model of AF. **a** Quantitative real-time RT-PCR (qRT-PCR) verification of the miRNA expression profile in pacing rabbits. **b** Representative Northern blot depicts the expression of miR-21 mRNA. **c** Mean miR-21 expression levels in the control group (*CR*), sham group (*SH*), and rapid atrial pacing group (*RAP*) groups. RAP caused a significant increase in miR-21 expression. **d** Quantitative analysis of TGF-β_1_ and Smad7 expression by real-time RT-PCR. The relative TGF-β_1_/Smad7 mRNA levels (TGF-β_1_/GAPDH or Smad7/GAPDH in arbitrary units) were normalized to the expression levels of group CR and group SH and the relative expression levels (in fold expression) were calculated. **e** Representative Western blot gel depicting the levels of TGF-β_1_ and Smad7 protein. **f** Mean TGF-β_1_ and Smad7 protein levels in the control group (CR), sham group (SH), and RAP group (RAP) groups. RAP caused a significant increase in expression of TGF-β_1_, but decreased the expression of Smad7 (*p < 0.05 vs. CR, unpaired *t* test; *n* ≥ 9 independent samples for each group)

As shown in Fig. 1a, qRT-PCR analysis confirmed that miR-21 was elevated by 4.5 times in RAP samples over control samples (Fig. 1a). The expression of endogenous miR-21 in the heart and the up-regulation of miR-21 were verified with Northern blot analysis (Fig. 1b, c). These findings prompted us to focus on exploring the role of miR-21 in AF and the associated atrial remodeling. Real-time PCR and quantitative Western blot analysis found that the expression of TGF-β_1_ mRNA and protein (Fig. 1d–f) was significantly higher in the left atrium of group RAP than with the non-paced group CR and group SH. However, the expression of the inhibitory Smad, Smad7, was significantly lower in the RAP group (Fig. 1e, f). This finding further confirmed that TGF-β_1_/Smad7 was involved in RAP-induced myocardial remodeling. Furthermore, through the computational prediction of target genes, we identified Smad7 as a potential target for miR-21. These findings prompted us to focus on exploring the role of miR-21 in AF and the association with Smad7 expression.

### Down-expression of miR-21 ameliorates myocardial fibrosis in vivo

**Fig. 2:**
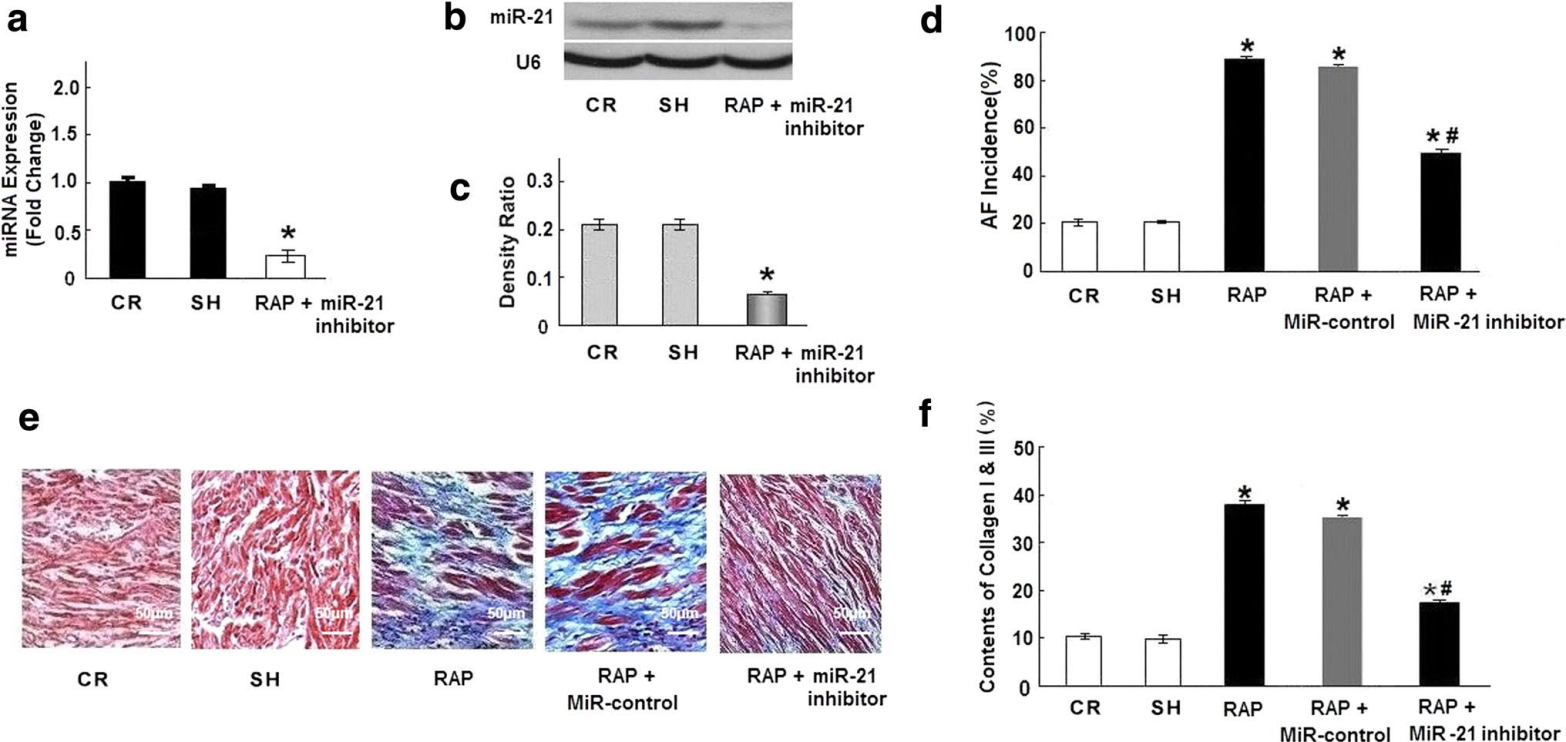
The effect of a miR-21 inhibitor in RAP-induced myocardial fibrosis in vivo. **a** miR-21 mRNA levels were examined by qRT-PCR. The miR-21 inhibitor decreased miR-21 expression in the heart of experimental rabbits (rabbits were treated with the miR-21 inhibitor and RAP for 4 weeks). **b** Representative Northern blot showing miR-21. **c** Mean miR-21 expression levels in the CR group and the RAP+miR-21 inhibitor group. **d** Mean incidence of AF. In contrast to the RAP group (89 % incidence of AF), treatment with the miR-21 inhibitor decreased the incidence of AF to 50 % (*p < 0.05). **e** Masson trichrome staining of rabbit atrial samples from the CR, RAP, and RAP+miR-21 inhibitor groups. The myocardium was stained red and collagens were stained blue. *Bar* 50 μm. **f** Mean collagen content in the left atria of the 3 groups (*n* ≥ 9 per group). RAP caused significant collagen deposition in the left atria that was attenuated by the miR-21 inhibitor (*p < 0.05 vs. CR, ^#^ *p* < 0.05 vs. RAP, unpaired *t* test; *n* ≥ 9 independent samples for each group)

On the basis of the above findings, we decided to examine whether reduced expression of miR-21 ameliorates myocardial fibrosis in AF. First, miR-21 inhibitor lentiviral vectors were injected into the jugular vein of rabbits 2 times per week for 4 weeks. RAP began one day after the injections and lasted for 4 weeks. At the end of the experiment, we found that miR-21 expression was significantly lower in the hearts of treated animals (Fig. 2a–c, *p* < 0.05), suggesting that miR-21 inhibitor lentiviral vectors can inhibit miR-21 expression in the heart. Next, we found that the incidence of atrial fibrillation after transfection with the miR-21 inhibitor lentiviral vector decreased from 89 % in the RAP group to 50 % in the lentiviral group (8 of 9 in the RAP group, 5 of 10 in the RAP+miR-21 inhibitor group). Furthermore, as shown in Table 1 and Fig. 2e, f, RAP increased the hydroxyproline content, collagen I and collagen III deposition, left atrial weight, and left atrial mass index of the animals’ hearts (Table 1). Transfection with the miR-21 inhibitor caused a significant decrease in hydroxyproline content, collagen I and collagen III deposition, left atrial weight, and left atrial mass index and attenuated the progression of RAP-induced atrial fibrosis. These data suggest that RAP-induced atrial fibrosis may be ameliorated by blocking miR-21 in the heart.

**Table 1:** RAP-induced hydroxyproline production, and LAW, and LAMI

Parameter	CR	SH	RAP	RAP + miR-21 inhibitor
Hydroxyproline	3.24 ± 0.31	3.26 ± 0.28	6.69 ± 0.47*	3.96 ± 0.25^**#**^
Body weight (g)	2.5 ± 0.2	2.4 ± 0.3	2.4 ± 0.3	2.4 ± 0.5
LAW (mg)	0.74 ± 0.15	0.76 ± 0.11	1.12 ± 0.27*	0.85 ± 0.17^**#**^
LAMI (mg/g)	0.31 ± 0.08	0.30 ± 0.07	0.52 ± 0.12*	0.40 ± 0.09^**#**^

Data are mean ± SE (*n* ≥ 9 for each group)

*LAMI* left atrial mass index, *LAW* left atrial weight

* *P* < 0.05 vs group CR; ^#^ *P* < 0.05 vs group RAP

**Fig. 3:**
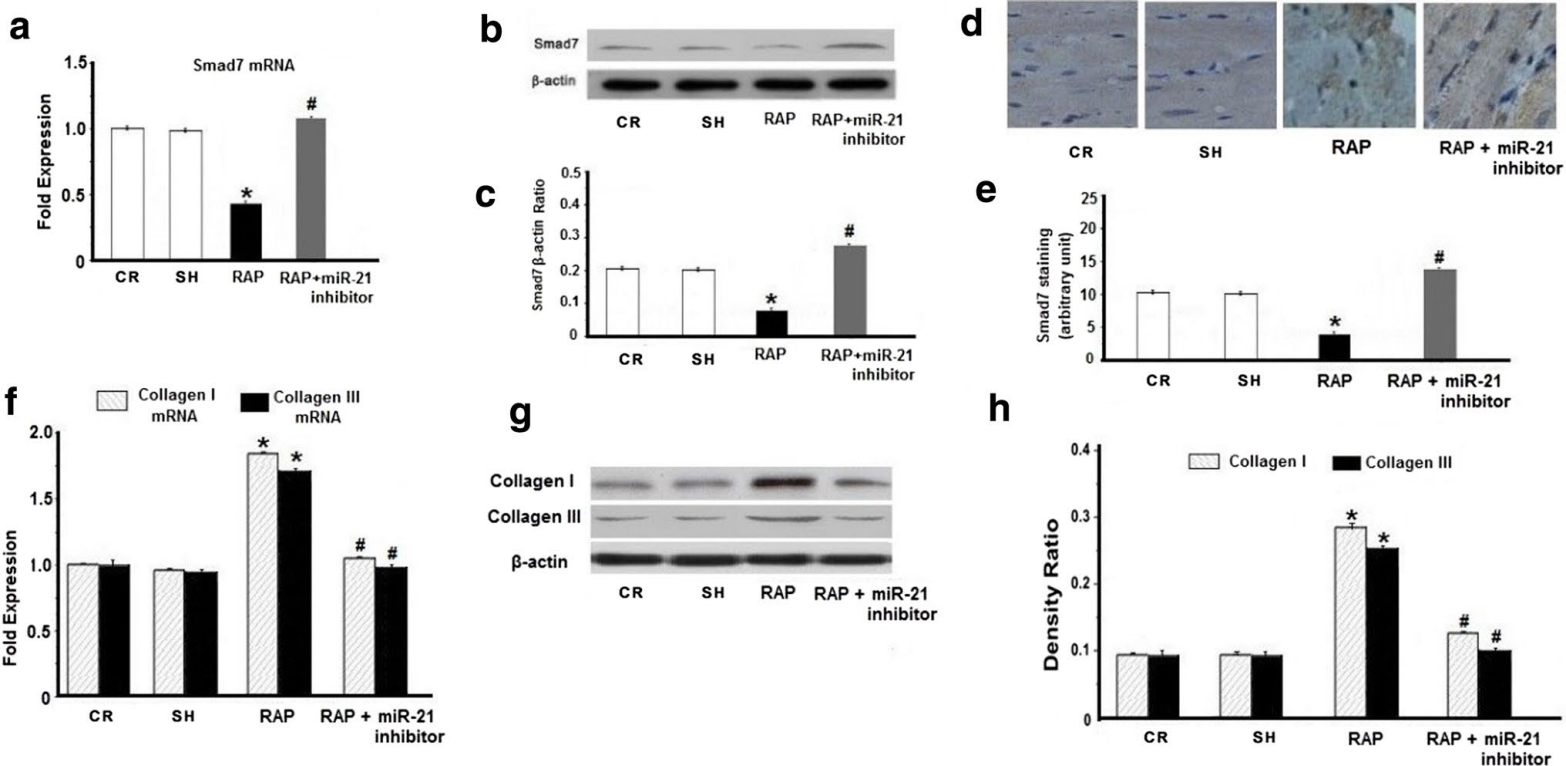
Effect of the miR-21 inhibitor on RAP-induced Smad7 and collagen I/III expression in vivo. **a** Quantitative analysis of Smad7 expression by qRT-PCR. The relative Smad7 mRNA expression ratio (Smad7/GAPDH in arbitrary units) in the left atria was normalized to the expression in group CR, and the relative expression levels (in fold expression) were calculated. **b** Representative Western blot depicts the expression of Smad7 protein. **c** Mean Smad7 protein levels in the CR, RAP and RAP + miR-21 inhibitor groups. The level of Smad7 was significantly lower in the left atria of the RAP group. After treatment with the miR-21 inhibitor, the level of Smad7 increased significantly compared with the CR group and the RAP group. **d** Representative immunohistochemical staining images for Smad7 (magnification: ×400). **e** Graphical representation of the abundance of Smad7 protein in the sections. **f** Quantitative analysis of the expression of collagen I and III transcripts by qRT-PCR. The relative collagen I and III mRNA levels (collagen I and III/GAPDH in arbitrary units) in the left atria were normalized to the levels in group CR and the relative expression levels (in fold expression) were calculated. **g** Representative Western blot depicts the expression of collagen I and III protein. **h** Mean collagen I and III protein levels in the CR, RAP and RAP + miR-21 inhibitor groups. The collagen I and III level was significantly higher in the left atria of the RAP group. After treatment with the miR-21 inhibitor, the level of collagen I and III decreased significantly compared with the CR and RAP group (*p < 0.05 vs. CR; ^#^ *p* < 0.05 vs. RAP, *n* ≥ 9 independent protein samples for each group)

Next, Smad7 and collagen I/III mRNA and protein were detected by qRT-PCR and Western blotting. Smad7 mRNA and protein levels were significantly lower than the CR group (Fig. 3a–c, *p* < 0.05). Conversely, collagen I/III mRNA and protein were significantly higher in the RAP group than the CR group (Fig. 3f–h, *p* < 0.05). Furthermore, immunohistochemical staining indicated that RAP induced the down-regulation of Smad7 (Fig. 3d, e, *p* < 0.05). More importantly, Smad7 expression increased after transfection with miR-21 inhibitor, whereas collagen I/III decreased (Fig. 3, *p* < 0.05). These data show that the miR-21 inhibitor could inhibit myocardial fibrosis by up-regulating Smad7 expression in vivo.

### Effect of TGF-β_1_ on miR-21 expression in vitro

Earlier reports have shown that miR-21 is extensively involved in myocardial fibrosis, and miR-21 is considered to be associated with TGF-β_1_-induced myocardial fibrosis [22, 32]. However, there are few reports about the role of miR-21 in AF. We have reported that TGF-β_1_ is up-regulated and promotes myocardial fibrosis in AF. In this study, we explored the relationship between miR-21 and TGF-β_1_ in cardiac fibroblasts.

**Fig. 4:**
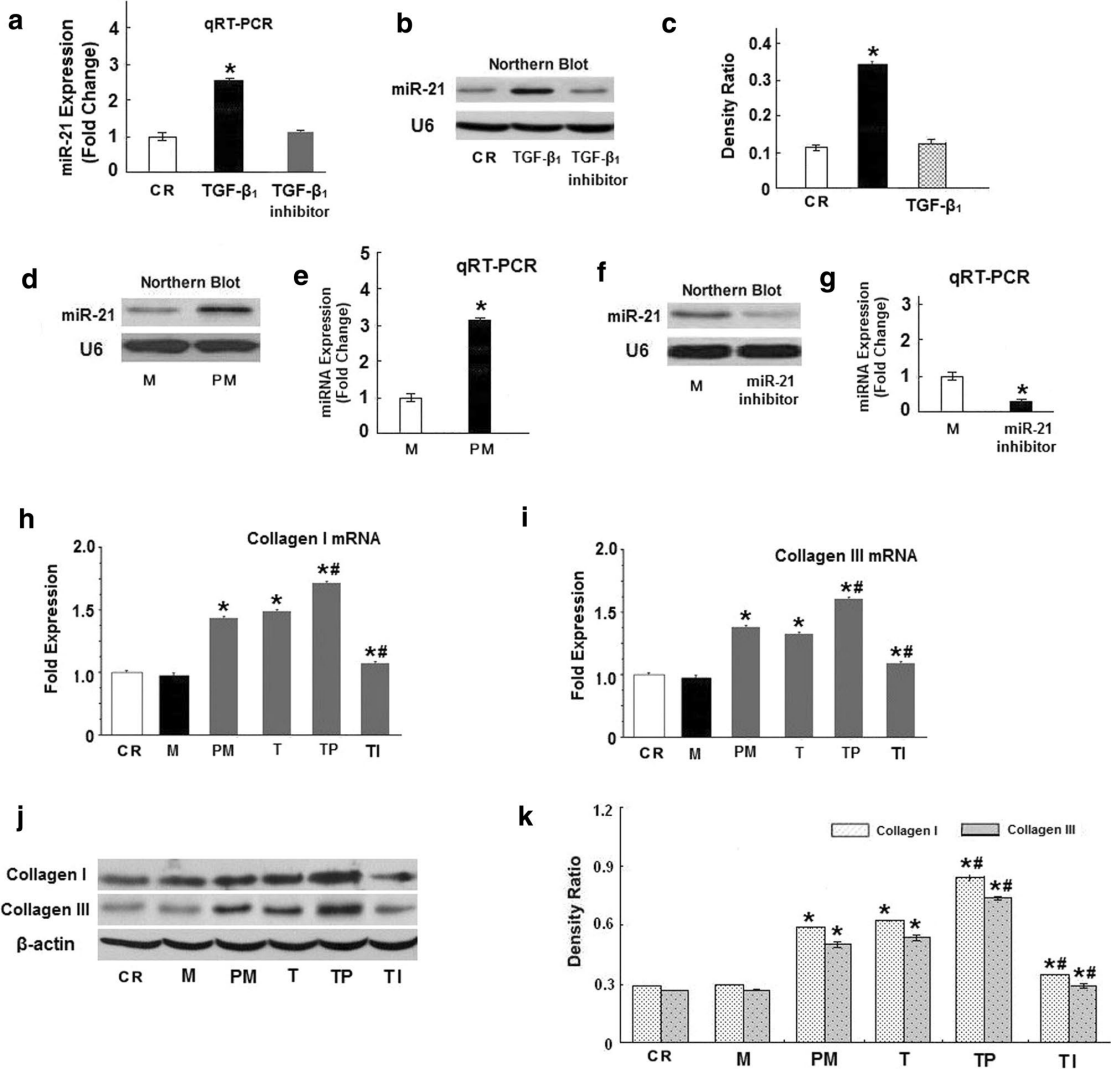
Effect of miR-21 on TGF-β_1_-induced collagen I/III expression. **a** qRT-PCR demonstrating that TGF-β1 (10 ng/ml) and TGF-β1 inhibitor(10 μg/ml) induces and decrease miR-21 expression at 48 h. **b** Representative Northern blot showing miR-21. **c** Values of miR-21 expression are the mean ± SE. **d** Northern blot verifying the upregulation of miR-21 in rats fibroblast after transfection with pre-miR-21. **e** qRT-PCR demonstrating that miR-21 levels increased in rat fibroblasts after transfection with pre-miR-21. **f** Northern blot verifying the down-regulation of miR-21 in rat fibroblasts after transfection with the miR-21 inhibitor. **g** qRT-PCR demonstrating that miR-21 levels deceased in rat fibroblasts after transfection with the miR-21 inhibitor. **h**, **i** Quantitative analysis of collagen I and III expression by qRT-PCR. The relative collagen I and III mRNA level (collagen I and III/GAPDH in arbitrary units) in the left atria was normalized to the expression in group CR and the relative expression levels (in fold expression) were calculated. **j** Representative Western blot depicting he expression of collagen I and III protein. **k** Mean collagen I and III levels in the control (*CR*), miR-control (*M*), pre-miR-21 (*PM*), TGF-β_1_ (*T*), TGF-β_1_ + pre-miR-21 (*TP*) and TGF-β_1_ + miR-21 inhibitor (*TI*) groups (*p < 0.05 vs. CR, ^#^ *p* < 0.05 vs. T, unpaired *t* test; *n* = 10 independent samples for each group)

First, miR-21 expression in the presence of TGF-β_1_ (10 ng/ml) was examined by qRT-PCR and Northern blot. We found that TGF-β_1_ can enhance miR-21 expression (Fig. 4a–c, *p* < 0.05). Next, we investigated whether miR-21 over-expression promoted the TGF-β_1_-induced expression of collagen I/III, which were detected by qRT-PCR and Western blot. Before treating the cells with TGF-β_1_, CFs were transfected with miR-control and miR-21 over-expression lentiviral vectors. miR-21 over-expression increased collagen I/III mRNA and protein levels. Meanwhile, CFs were treated with TGF-β_1_, with the results indicating that TGF-β_1_ could increase collagen I/III mRNA expression (Fig. 4h, i). Next, CFs were transfected with miR-control and miR-21 over-expression lentiviral vectors before being transfected with TGF-β_1_ (10 ng/ml) for 48 h. TGF-β_1_ plus miR-21 overexpression enhanced collagen I/III mRNA expression more significantly than TGF-β_1_ treatment alone (Fig. 4h, i). Interestingly, we found that miR-21 inhibition significantly weakened TGF-β_1_-induced collagen I/III mRNA expression in the CFs that were treated with TGF-β_1_ plus the miR-21 inhibitor. Furthermore, the effect on collagen I/III proteins was consistent with the effect on these mRNAs (Fig. 4j, k). Taken together, our results demonstrate that TGF-β_1_-induced collagen I/III expression might result from the expression of miR-21 in vitro.

### Over-expression of TGF-β_1_ induces Smad7 down-regulation through miRNA-21

**Fig. 5:**
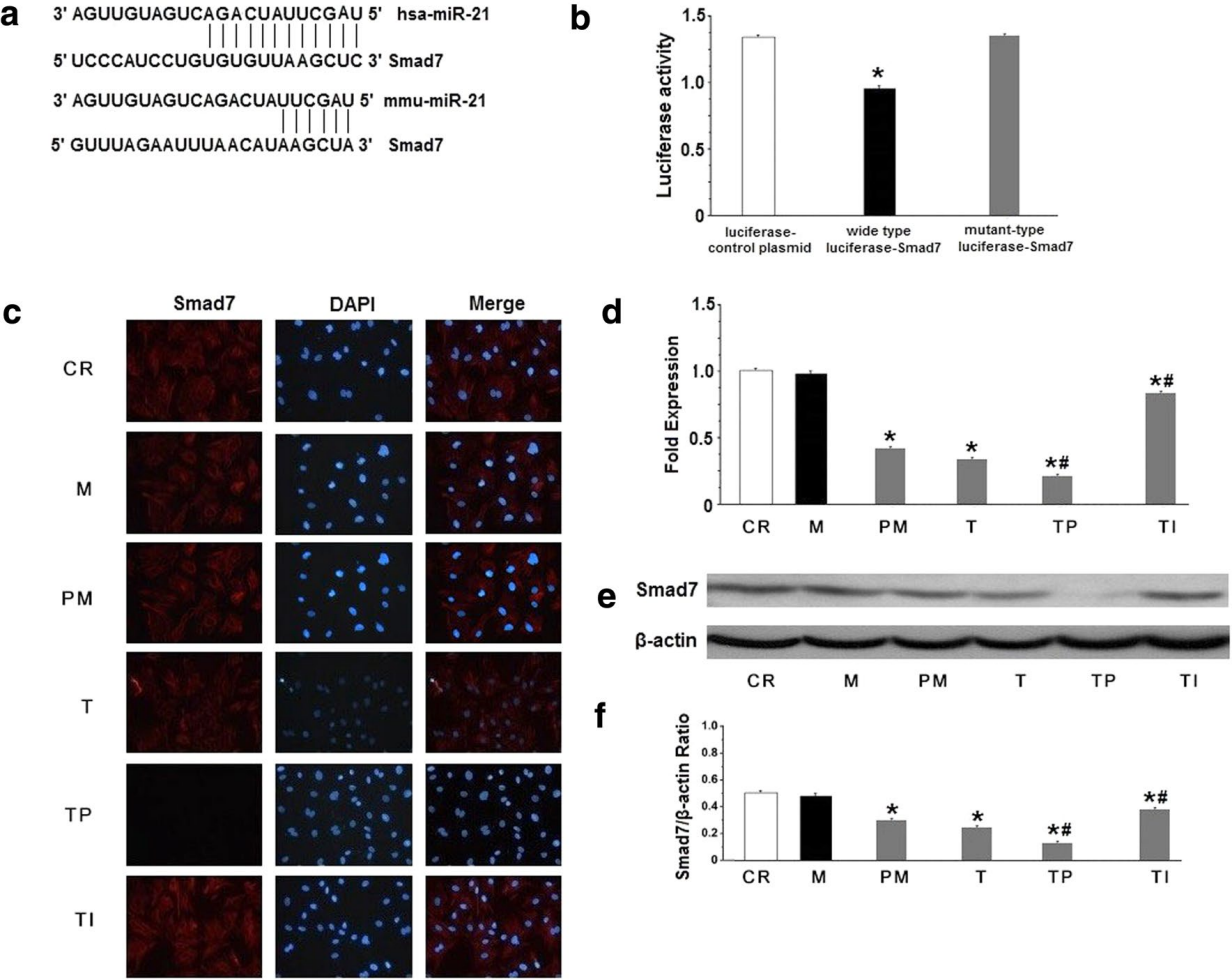
Effect of miR-21 on the TGF-β_1_-mediated down-regulation of Smad7. **a** Alignment of hsa-miR-21 and mmu-miR-21 with human Smad7 3′-UTR and mouse Smad7 3′-UTR based on TargetScan and Pictar software (http://www.targetscan.org/ and http://pictar.mdcberlin.de/). Several nucleotides in the 50-region of miR-21 (human and mouse) contain a perfect match with the 3′-UTR sequences of the human and mouse Smad7 genes. **b** The results of luciferase reporter assays. **c** Representative immunocytochemical images of Smad7 and propidium iodide (*PI*) staining. Cardiac fibroblasts were treated with TGF-β_1_ in the presence or absence of miR-21 for 48 h. **d** Quantitative analysis of Smad7 expression by qRT-PCR. The relative Smad7 mRNA expression ratio (Smad7/GAPDH in arbitrary units) was normalized to that of group CR and the relative expression levels (in fold expression) were calculated. **e** Representative Western blot depicting the expression of Smad7 protein. **f** Mean Smad7 protein levels in the control (*CR*), miR-control (*M*), pre-miR-21 (*PM*), TGF-β_1_ (*T*), TGF-β_1_ + pre-miR-21 (*TP*) and TGF-β_1_ + miR-21 inhibitor (*TI*) groups (*p < 0.05 vs. CR, ^#^ *p* < 0.05 vs. T, unpaired *t* test; *n* = 10 independent samples for each group)

Our results indicated that TGF-β_1_-induced collagen I/III expression might occur through the up-regulation of miR-21, meanwhile we found in our previous studies that the over-expression of TGF-β_1_ induced the degradation of Smad7 which may decrease the inhibitory feedback regulation of TGF-β_1_/Smad signaling pathway, so we sought to determine the relationship between miR-21 and Smad7 in TGF-β_1_-induced collagen expression. According to the TargetScan (http://www.targetscan.org/) and Pictar databases (http://pictar.mdcberlin.de/) and previous experiments, Smad7 is a validated target of miR-21 [25, 33]. Therefore, to further confirm that Smad7 is a validated miR-21 target in CFs, we performed luciferase reporter assays. The expression of the wild-type luciferase-Smad7-3′UTR reporter was remarkably lower than the mutant luciferase-Smad7-3′UTR reporter and control plasmid (Fig. 5a, b), suggesting that Smad7 is a validated miR-21 target in CFs and that miR-21 can inhibit Smad7 expression, leading to the further amplification of TGF-β_1_ signaling. Next, we investigated the effect of miR-21 over-expression on Smad7 mRNA and protein, which were detected by RT-PCR and western blot. Before treatment with TGF-β_1_, CFs were transfected with the miR-control and miR-21 over-expression lentiviral vectors. In this assay, we found that miR-21 over-expression decreased Smad7 mRNA levels. Next, we tested the effect of miR-21 overexpression on TGF-β_1_-induced Smad7 down-regulation. Prior to the transfection, we confirmed that TGF-β_1_ decreased Smad7 mRNA expression in CFs. Next, TGF-β_1_-treated CFs were transfected with the miR-control and miR-21 over-expression lentiviral vectors. The results of this assay demonstrated that miR-21 over-expression significantly enhanced TGF-β_1_-induced Smad7 down-regulation compared with the TGF-β_1_ group and the miR-control group (Fig. 5d). Interestingly, we found that miR-21 inhibition significantly weakened TGF-β_1_-induced Smad7 mRNA down-regulation in the CFs that treated with TGF-β_1_ plus the miR-21 inhibitor (Fig. 5d). Moreover, the effect on the Smad7 proteins was in line with the effect on these mRNAs (Fig. 5e, f). Immunocytochemistry showed that TGF-β_1_ decreased the nuclear and perinuclear accumulation of Smad7. The miR-21 inhibitor, but not pre-miR-21, increased the accumulation of Smad7 (Fig. 5c). Taken together, our data show that miR-21 over-expression directly down-regulates Smad7 expression.

## Discussion

In this study, we examined the relationship between miR-21 and TGF-β_1_/Smad7 signaling in AF-induced atrial fibrosis. The major findings of this study include the following: (1) our real-time PCR and quantitative Western blot analyses revealed that RAP-induced AF significantly down-regulated Smad7 expression, which was associated with an increase in TGF-β_1_ and collagen I/III; (2) the AF-induced up-regulation of miR-21 was negatively correlated with Smad7 expression; (3) in isolated adult rat cardiac fibroblasts, treatment with TGF-β_1_ caused the down-regulation of Smad7 expression and increased the levels of miR-21 and collagen I/III; (4) the luciferase reporter assays suggested that Smad7 is a validated miR-21 target in CFs; and (5) in the presence of TGF-β_1_, inhibiting or upregulating miR-21 could increase and decrease Smad7 expression, respectively, indicating that the TGF-β_1_-induced decrease in Smad7 expression might be mediated by miR-21. These results strongly suggest that the miR-21-mediated degradation of Smad7 might decrease the inhibitory feedback regulation of TGF-β1/Smad signaling and serves as a new insight into the development of atrial fibrosis due to atrial fibrillation.

### AF and atrial fibrosis

Several animal models of chronic sustained AF have been developed to assess the atrial electric remodeling and arrhythmias of AF. In AF, atrial effective refractory period (AERP) was decreased, and the animals became more vulnerable to AF [4, 34]. It has been reported that rapid pacing induces AF with much higher incidence at the left atrial site than at the right atrium or any other sites, partly because of the inhomogeneous dispersion of AERP. When atrial electrical remodeling returns back to normal, the underlying reasons for persistent AF and structural remodeling are still present. Thus, atrial electrical remodeling alone is sufficient to induce AF, while structural remodeling is responsible for maintaining AF [35, 36]. In this study, we have successfully established the electrical stimulation rapid pacing left atrium-induced AF model. We demonstrated that RAP alone induced profound changes in gene expression of collagens. In addition to the increase in the hydroxyproline content and left atrial weight, a significant deposition of cardiac collagen I and III was observed in the atria subjected to rapid pacing.

### TGF-β1 induces myocardial fibrosis by upregulating miR-21

Tachycardia-induced atrial fibrosis is a hallmark of AF-induced structural remodeling. This condition is characterized by the accumulation of extracellular matrix (ECM) and myocardial fibrosis [10, 37]. In this study, we demonstrated that RAP alone induced profound changes in the expression of collagen I/III in the heart, strongly suggesting that tachycardia during AF might cause atrial remodeling resulting from atrial fibrosis. In addition to the increase in hydroxyproline content and left atrial weight, a significant deposition of cardiac collagen I and III was observed in the atria subjected to rapid pacing. These results also support our previous observations that atrial fibrosis consisting of collagen types I and III is a typical feature of AF.

Increasing evidence indicates that targeting TGF-β_1_/Smad-specific miRNAs related to fibrosis may be a better approach for combating heart disease [38–40], with miR-21 being the best characterized miRNA associated with TGF-β_1_-mediated fibrosis [41]. It reported that miR-21 is expressed much higher in cardiac fibroblasts and accompanied by the increased expression of collagen in pathological states of heart failure [42]. In a rat model of myocardial infarction, the down-regulation of miR-21 can reduce the degree of atrial fibrosis and the incidence of AF [43]. Adam recently confirmed miR-21 levels were significantly higher in patients with atrial fibrillation, and the up-regulation of miR-21 target genes can increase fibrin deposition [19]. We have observed previously that RAP may cause atrial fibrosis through the AngII/AT1 receptor–specific activation of the TGF-β_1_/Smad pathway and that TGF-β_1_ overexpression induces myocardial fibrosis in AF [10]. However, whether miR-21 over-expression enhanced TGF-β_1_-induced myocardial fibrosis in AF remained elusive. In vivo, we found that RAP induced TGF-β_1_ and miR-21 up-regulation with the increased expression of collagen. Transfection with the miR-21 inhibitor, we found it reduce the incidence of AF and ameliorated RAP-induced atrial fibrosis. In vitro, we found that miR-21 over-expression increased collagen I/III expression and that miR-21 over-expression after treatment with TGF-β_1_ further increased collagen I/III expression. In addition, blocking miR-21 expression reverses the TGF-β_1-_induced up-regulation of collagen I/III. Thus, our study further suggested that miR-21 may be involved in the regulation of RAP-induced myocardial fibrosis, and we speculate that miR-21 over-expression significantly enhances TGF-β_1_-induced collagen expression.

### Mechanisms underlying AF-induced down-regulation of Smad7 expression

The down-regulation of Smad7 may be key in the increased activation of the TGF-β_1_/Smad signaling pathway that is responsible for increased atrial fibrosis during AF [10, 44]. Smad7 not only exhibits a protective role in Ang II-induced cardiac fibrosis and inflammation, but also possesses therapeutic potential for hypertensive cardiac disease [11, 16]. In our previous study, we reported that the overexpression of Smad7 blocked AngII-induced increases in collagen I synthesis and that the overexpression of TGF-β_1_ decreased Smad7 expression [10]. In this study, we also found that RAP decreased Smad7 expression, which is negatively correlated with TGF-β_1_ and collagen I/III expression. This finding further confirmed that the down-regulation of Smad7 might cause myocardial fibrosis in AF. miR-21 is the best characterized miRNA associated with TGF-β_1_-mediated fibrosis [41]. For example, miR-21 and Smad7 are critical regulators of TGF-β_1_ signaling during the induction of carcinoma-associated fibroblast formation [33, 45, 46]. miR-21 overexpression enhances the TGF-β_1_-induced epithelial-to-mesenchymal transition by targeting Smad7 and aggravates renal damage in diabetic nephropathy. However, the regulation mechanism linking miR-21 and Smad7 in RAP-induced myocardial fibrosis was unclear. To further demonstrate our observations in this study and gain more information about how Smad7 is down-regulated during RAP, we examined growth-arrested adult rat fibroblast cells. Moreover, bioinformatics and the luciferase reporter assays showed that Smad7 is a validated target of miR-21. We found that miR-21 over-expression decreases Smad7 expression, which was similar to the change that occurred with TGF-β_1_ treatment. Furthermore, miR-21 over-expression significantly enhanced TGF-β_1_-induced Smad7 down-regulation. Interesting, inhibiting miR-21 expression weakened the TGF-β_1_-mediated down-regulation of Smad7 expression. This study further confirmed our previous research that the down-regulation of Smad7 may be responsible for RAP-induced myocardial fibrosis. More interestingly, the molecular mechanism underlying this phenomenon is as follows: the expression of TGF-β_1_ leads to miR-21 up-regulation, and miR-21 over-expression directly down-regulates Smad7 expression, which leads to amplification of TGF-β_1_ signaling and ultimately results in RAP-induced fibrosis. Thus, we speculate that miR-21 over-expression significantly enhances TGF-β_1_-induced collagen expression and that Smad7 can directly inhibit collagen expression in RAP-induced myocardial fibrosis.

In summary, our data demonstrate that TGF-β_1_-induced miR-21 over-expression enhances TGF-β_1_-induced collagens expression by directly down-regulating Smad7. These results may provide new insights into the mechanisms underlying AF-induced atrial fibrosis and myocardial remodeling and provide valuable information for novel therapeutic targets for AF.

## Electronic supplementary material

Below is the link to the electronic supplementary material.

Supplementary material 1 (DOCX 14 kb)
