# Supplementary material for: Rapid atrial pacing induces myocardial fibrosis by down-regulating Smad7 via microRNA-21 in rabbit
Source: Heart Vessels. 2016 Mar 11;31(10):1696–708. doi: 10.1007/s00380-016-0808-z (PMC5043001; doi:10.1007/s00380-016-0808-z)
Supplement: Supplementary file 1 — Supplementary material 1 (DOCX 14 kb) [file 380_2016_808_MOESM1_ESM.docx]

The electrophysiology data.

|  | Before RAP | | After RAP | | | |
| --- | --- | --- | --- | --- | --- | --- |
|  |  | | AERP(ms) | | | |
| Group | PR(ms) | QRS(ms) | PR(ms) | QRS(ms) | BCL 200ms | BCL 120ms |
| CR | 52±4 | 48±5 | 53±4 | 48±4 | 108±4 | 92±4 |
| SH | 53±5 | 47±4 | 54±4 | 47±3 | 107±4 | 93±3 |
| RAP | 54±3 | 48±4 | **…** | 48±3 | 80±3* | 69±2* |
| RAP+ miR-control | 53±6 | 47±5 | 54±3 | 47±4 | 82±4* | 70±3* |
| RAP+  miR-21 inhibitor | 53±5 | 47±6 | 54±3 | 48±4 | 104±3**^#^** | 90±2**^#^** |

*P<0.05 vs group CR; ^#^P<0.05 vs group RAP.
